# Changes in Lifestyle, Behaviors, and Risk Factors for Cognitive Impairment in Older Persons During the First Wave of the Coronavirus Disease 2019 Pandemic in Finland: Results From the FINGER Study

**DOI:** 10.3389/fpsyt.2021.624125

**Published:** 2021-02-12

**Authors:** Jenni Lehtisalo, Katie Palmer, Francesca Mangialasche, Alina Solomon, Miia Kivipelto, Tiia Ngandu

**Affiliations:** ^1^Department of Neurology, Institute of Clinical Medicine, University of Eastern Finland, Kuopio, Finland; ^2^Population Health Unit, Finnish Institute for Health and Welfare, Helsinki, Finland; ^3^Department of Geriatrics, Centro Medicina dell'Invecchiamento, Università Cattolica del Sacro Cuore, Rome, Italy; ^4^Division of Clinical Geriatrics, Department of Neurobiology, Center for Alzheimer Research, Care Sciences and Society, Karolinska Institutet, Stockholm, Sweden; ^5^Department of Neurobiology, Aging Research Center, Care Sciences and Society, Karolinska Institutet and Stockholm University, Stockholm, Sweden; ^6^The Ageing Epidemiology Research Unit, School of Public Health, Imperial College London, London, United Kingdom; ^7^Theme Aging, Karolinska University Hospital, Stockholm, Sweden; ^8^Institute of Public Health and Clinical Nutrition, University of Eastern Finland, Kuopio, Finland

**Keywords:** COVID-19, SARS-CoV-2 (CoVID-19), quarantine, non-communicable diseases, lifestyle, prevention, cognitive impairment, aging

## Abstract

**Aims:** This study aimed to describe how the first phase of the coronavirus disease 2019 (COVID-19) pandemic affected older persons from the general Finnish population who are at risk of developing or have cognitive impairment, specifically, to describe whether participants experienced a change in risk factors that are relevant for the prevention of cognitive decline including diet, physical activity, access to medical care, socially and cognitively stimulating activities, and emotional health and well-being.

**Method:** A postal survey was sent in June 2020 to 859 participants from the Finnish Geriatric Intervention Study to Prevent Cognitive Impairment and Disability (FINGER), an ongoing longitudinal study. The survey was developed to assess the effect of the COVID-19 pandemic and related infection-control measures on daily life, specifically commitment to distancing measures, access to health care and social services, daily activities, and changes in cognitive and social activities.

**Results:** By September 2020, 613 (71%) participants responded (mean age = 77.7 years, 32% lived alone, and 80% had at least one chronic condition). Three quarters adopted some distancing practices during the first months of the pandemic. Older participants were more likely to practice total isolation than younger ones (29 vs. 19%; *p* = 0.003). Non-acute health-care visits were canceled for 5% of the participants who needed appointments, but cancellations in dental health care (43%), home aid (30%), and rehabilitative services (53%) were more common. Pandemic-related changes were reported in social engagements, for example, less contact with friends (55%) and family (31%), or less frequent attendance in cultural events (38%) or associations (25%), although remote contact with others increased for 40%. Feelings of loneliness increased for 21%, particularly those who were older (*p* = 0.023) or living alone (*p* < 0.001). Physical activity reduced for 34%, but dietary habits remained stable or improved. Pandemic-related changes in lifestyle and activities were more evident among those living alone.

**Conclusions:** Finnish older persons generally reported less negative changes in lifestyles and behaviors during the pandemic than expected. Older people and those living alone seemed more susceptible to negative changes. It is important to compare how coping strategies may compare with other European countries to identify factors that may help older individuals to maintain healthy lifestyles during future waves of COVID-19.

## Introduction

Multidomain lifestyle interventions targeted at community-dwelling older persons, such as the Finnish Geriatric Intervention Study to Prevent Cognitive Impairment and Disability (FINGER) study (1), have shown that multiple aspects of health (e.g., diet, exercise, cognitive training, and metabolic/vascular risk monitoring) are important for reducing the risk of cognitive decline. Recent guidelines from the World Health Organization (WHO)^1^ for reducing the risk of cognitive decline and dementia emphasize the need to control vascular and metabolic risk factors and lifestyle-related factors. Many of these risk factors are common to other noncommunicable diseases (NCDs) (2), and indeed, the FINGER study reported that multidomain interventions can also help to prevent or delay other negative health outcomes, including decline in physical functioning and multimorbidity over 2 years of follow-up (3, 4). In light of the restrictions enforced in many counties during the coronavirus disease 2019 (COVID-19) pandemic to control the risk of severe acute respiratory syndrome coronavirus 2 (SARS-CoV-2) infection, we must consider if these initiatives have a short- and/or long-term effects on risk factors for NCDs and cognitive impairment, especially in older individuals. It has been hypothesized that changes in diet, levels of physical activity, cognitive and social stimulation, and access to routine NCD management may occur in some individuals during the pandemic and that this may affect their long-term health (5), potentially altering their risk of developing NCDs in the future. Surveys conducted during the first wave of the pandemic have reported reduced physical activity, dietary changes, and disruptions to NCD care, among others, in various countries (6–12). However, as each country applied varying strategies to contain the spread of the COVID-19 virus, information is needed concerning how these initiatives have affected persons living in different countries.

As of 23 October 2020, there were 14,474 confirmed cases of COVID-19 in Finland^2^, with the first reported cases occurring on 29 January 2020 and a peak of COVID-19-related deaths occurring in mid-April^3^. On 16 March 2020, the Emergency Powers Act was implemented, with decisions to suspend contact teaching; limitations to public gatherings; closure of public services such as museums, libraries, and sports facilities; ban of visitors to care institutions and hospitals; instructions to work remotely; reduction of non-acute health and social services; and further travel restrictions. A strong but not compulsory guideline for persons over 70 years of age was given in that they must refrain from contact with other persons to the extent possible (quarantine-like conditions). Some restrictions were gradually lifted during May and June 2020, including opening of, first, outdoor, and, then, indoor recreational facilities. On 23 June, the age-based strong recommendation to avoid personal contact was lifted.

Research into the effects of the COVID-19 pandemic and associated infection-control measures is ongoing in many countries. Ongoing population-based longitudinal studies can provide important insight into how the pandemic has affected the general population: first, because they provide quick access to already established research participants and, second, because they provide pre-pandemic data on individuals' health and functioning to allow for accurate measures of change. The FINGER study (1, 13), described later, was a 2-year multidomain intervention aimed at delaying cognitive decline in community-dwelling persons aged 60–77 who were at risk of developing cognitive impairment or dementia. The study was initiated in 2009, and until now, participants have undergone a comprehensive follow-up evaluation to assess cognitive and health status at 2, 5, and 7 years of follow-up. A 10-year follow-up was planned in 2020 but was halted as a result of the COVID-19 pandemic. Within the context of the WORLDWIDE-FINGERS-SARS-COV-2 INITIATIVE of multidomain prevention trials (14), which is an initiative to test and adapt the FINGER intervention model in over 25 countries worldwide, we developed a postal survey to assess how COVID-19 and associated infection-control measures (such as quarantines and lockdowns) would affect participants in terms of changes in lifestyle, risk factors, social stimulation, and access to medical care. Preliminary data from the Finnish FINGER COVID-19 survey are now available.

The aim of the current study is to describe how the first phase of the COVID-19 pandemic affected older persons from the general Finnish population who are at risk of developing dementia. Previous studies suggest that, particularly, social isolation during the pandemic has negative impact on both physical and mental health (15). Specific objectives are to describe whether participants experienced a change in risk factors that are relevant for the prevention of cognitive decline, dementia, and other NCDs, including diet, physical activity, access to medical care (and, thus, opportunities for controlling vascular and metabolic risk factors), socially and cognitively stimulating activities, and emotional health and well-being.

## Methods

### Setting and Study Population

FINGER is a multidomain lifestyle intervention trial covering six areas in Finland (ClinicalTrials.gov NCT01041989). The study comprises a population-based sample recruited from previous national surveys. Participants were aged 60–77 years in the beginning of the study and had an elevated risk of developing dementia based on CAIDE dementia risk score (13, 16). They underwent screening with a short neuropsychological examination with the Consortium to Establish a Registry for Alzheimer's Disease (CERAD) test battery (17) and medical examination by a study physician. Participants with a CAIDE dementia risk score of 6 or higher were invited to the trial, if they were free of dementia and conditions affecting safe engagement in the intervention and had cognitive performance at average level or slightly below than expected for age. They were randomized 1:1 to multidomain lifestyle intervention or regular health advice. All participants in the multidomain intervention group received intervention in four domains: dietary counseling, exercise training, cognitive training, and management of cardiovascular and metabolic factors (13).

**Figure 1:**
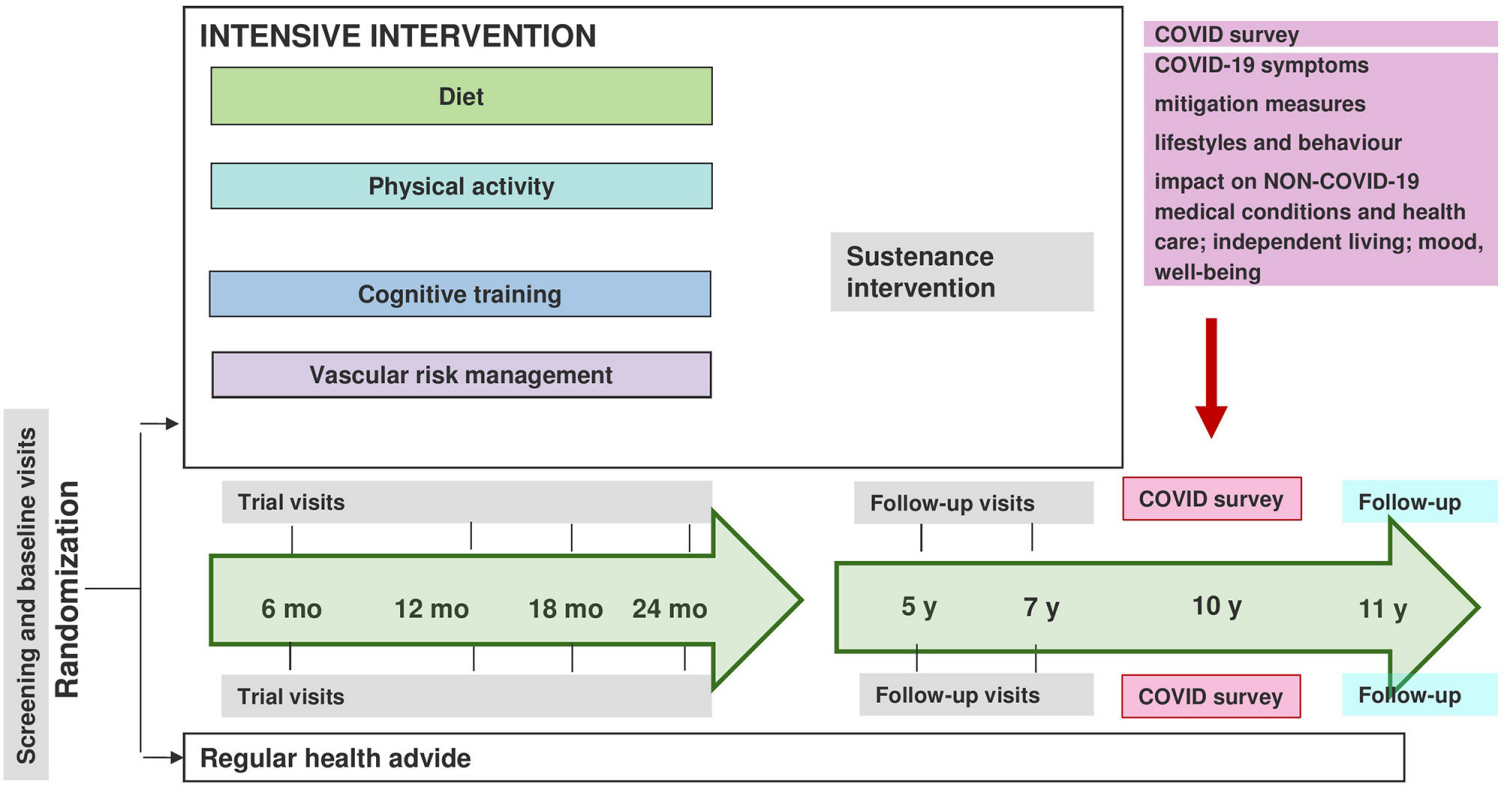
Overview of the Finnish Geriatric Intervention Study to Prevent Cognitive Impairment and Disability (FINGER) study setting.

The original intervention period lasted for 2 years for each participant (during 2009–2013), and post-intervention follow-up examinations have been conducted at 5 and 7 years (3 and 5 years after the intervention). A 10-year follow-up was planned to start in 2020, but when the COVID-19 outbreak emerged, face-to-face examinations were postponed. A specific survey with questions relating to the COVID-19 pandemic was developed (see details later), and participants were mailed with a questionnaire in June 2020, immediately following the strict restrictions initiated in Finland due to the first wave of COVID-19 (Figure 1).

A total of 859 participants from the original FINGER population (*n* = 1,259, 69%) were eligible for invitation to answer the questionnaire after those who had died (*n* = 182) or previously withdrawn from the study (*n* = 218) were excluded.

The survey is an amendment to the current FINGER protocol and was approved by the coordinating ethics committee of the hospital district for the Helsinki and Uusimaa region.

### COVID-19 Questionnaire

The questionnaire included questions about health, health-care use, lifestyles and daily living, quality of life, mood, and personality in relation to the COVID-19 outbreak. The survey is harmonized with the questionnaire devised within the WORLDWIDE-FINGERS-SARS-COV-2 INITIATIVE for later pooled analyses, and partly with the Finnish population-based survey^4^ conducted on all adult ages, run by the Finnish Institute for Health and Welfare, to enable the comparison of different age groups later.

In the current paper, we focus on describing the effect of the COVID-19 pandemic and related infection-control measures on daily life among older adults, specifically their commitment to distancing measures, access to health care and social services, daily living, and any relevant changes in cognitive and social activities. The survey provided information on the following characteristics:

1) Sociodemographic Characteristics

Age, education, and marital status were collected at FINGER baseline, and marital status again in the COVID-19 questionnaire. Age at the time of compiling the COVID-19 questionnaire was calculated based on dates in the questionnaires (or 1 July 2020 for comparing respondents with non-respondents). Marital status was dichotomized into those living with someone (married or cohabitation) vs. living alone (single, divorced, or widow). Information on housing was also collected. For age-group comparisons, age was grouped based on median value, that is, below or above 77.7 years.

2) Distancing Measures During the COVID-19 Pandemic

Participants were asked if they considered that they had followed a self-chosen isolation/quarantine (staying at home and nobody visiting); quarantine enforced by authorities; total social distancing (no shopping or running errands indoors, but possibly going out, e.g., for a walk); and partial social distancing (e.g., running essential errands or meeting people outdoors while keeping distance). Participants were allowed to select as many options as they thought necessary and to specify amount of weeks they followed each type of isolation since the beginning of the pandemic. They were also asked if they continued to practice some type of isolation at the time of completing the questionnaire.

We combined self-chosen and authority-enforced isolation into one group for reporting, and we calculated total time spent in total isolation. Furthermore, a total amount of time spent with some type of distancing measures, less strict than total isolation, was calculated. If the same participant reported not having adopted any distancing measures but still chose some of the options related to them, he/she was considered as having adopted distancing. As many participants had missing values or zero for weeks of the chosen distancing type, they were included when reporting numbers of people who practiced each specific type of distancing, but their time estimate was not taken into account when calculating the average duration.

3) Non-Acute Health-Care Usage During the COVID-19 Pandemic

Participants were asked to report any chronic condition that they had been diagnosed with from a list, including asthma, chronic obstructive pulmonary disease, other lung disease, diabetes or elevated blood glucose, high cholesterol, hypertension, heart disease (angina pectoris, coronary artery disease, previous heart attack or angioplasty or bypass surgery), cardiac failure, cancer, cancer treatment, epilepsy, mental health condition (e.g., depression, anxiety, etc.), cognitive impairment or memory disorder, cerebral hemorrhage or other cerebrovascular disease, multiple sclerosis, rheumatoid arthritis, renal failure, organ transplant, condition, and/or drugs that weaken the immune system. For each chosen condition, they were asked if they had had issues in getting treatment for that condition during the COVID-19 outbreak. The options were canceling an appointment themselves, having an appointment canceled by health-care professionals, having remote medical examinations (phone call or video call), having face-to-face appointments as usual, or not needing any medical care.

Access to dental health care, mental health care, social services, supportive services (e.g., home care or home aid), or rehabilitation services (e.g., physiotherapy or daytime activities) during the COVID-19 outbreak were also asked. Supportive or rehabilitation services were asked for the participant or for a close person from the same household. Participants were asked to choose if they had canceled a visit themselves, had a visit canceled by the professional, had visits/help face to face as usual, or not needed any visits/help. An option for remote contact was not provided in this question.

4) Changes in Daily Life, Lifestyle, and Emotional Health During the COVID-19 Pandemic and Related Periods of Infection-Control Measures

Participants were asked to evaluate how the COVID-19 pandemic and related restrictions have affected their experienced daily life. We included questions about time spent with family, contact with friends and relatives, experience of loneliness, experience of closeness with other people, family conflict, fear or experience of domestic violence or violence by a close relative, hopefulness for the future, leisure time physical activity, smoking, alcohol use, sleep problems and nightmares, number of meals and snacks per day, appetite, vegetable consumption (raw and cooked vegetables and salad excluding potato), fruit and berry consumption, snacking (sweets, chocolate, soft drinks, chips, biscuits, etc., consumption), internet use (e.g., smartphone, computer, and tablet), using digital services for everyday routines (e.g., ordering food online and online banking), using digital services or contact by phone for social and health-care services (e.g., speaking with a doctor or nurse), and remote contact with relatives and friends (messaging and video or phone calls). For each item, we asked if it was similar before the pandemic, decreased or increased, or not applicable/relevant to the participant. For the current analyses, no change and “not applicable” were merged to focus on changes experienced during the pandemic. Items for which decrease was considered a positive change (i.e., loneliness, discordance in the family, fear of violence, sleeping problem, smoking, alcohol consumption, and unhealthy snacks) were recoded to ease interpretation of results, and change in all items is categorized as no change, worsening, or improvement.

5) Changes in Engagement in Social and Cognitive Activities as a Result of the COVID-19 Pandemic or Related Infection-Control Measures

Social and cognitive activities were evaluated with the same questions that we used previously in the FINGER trial (13) and included questions concerning average frequency of reading, doing crosswords, writing, games, listening or playing music, communal activities or participation in societies, studying, handicrafts, gardening, looking after children (other people's; either family or friends), and voluntary work. Frequency of engagement in those activities was measured on a 7-point Likert scale with alternatives from daily to never (daily; four to six times per week; two to three times per week; once a week; two to three times per month; a few times per year; and never). The same items and same scale were presented twice; first, the participants were advised to evaluate their life before the pandemic and then during the pandemic. For the current analyses, we defined change as transition in frequency categories between the two timepoints and categorized as any decrease, the same frequency, or any increase. Any increase in activities was considered positive, and changes in these activities are referred as no change, worsening, or improvement.

6) Changes on Self-Rated Health and Quality of Life

Participants were asked to evaluate if their quality of life, physical condition, functional status, memory, or overall health had changed during the COVID-19 pandemic. Options for each question included better, worse, or similar compared with pre-pandemic time or “I do not know.” Similar and not being able to say were combined in these analyses. For memory, there were more options with “slightly” or “significantly” better and worse, which were merged to provide final options as no change, worse, or better.

### Statistical Methods

We report descriptive data as mean and standard deviation for continuous variables and counts and proportions for factor variables. Comparisons were executed between respondents and non-respondents for main characteristics, and between age groups and marital status for the COVID-19 questionnaire data. Comparisons were done using *t*-test or χ^2^-test, as appropriate. Analyses were executed with Stata/SE version 16.1.

## Results

The postal survey was sent out on 22 June 2020. Preliminary data are available for 613 participants (97% were living at home) who returned their questionnaires by post before 1 September 2020 (one empty questionnaire returned, total *n* = 614), after which a second questionnaire was mailed to non-respondents (collection ongoing). Participants from the original FINGER study who were included in the current sample were younger: their mean age was 68.2 (SD 4.7) at baseline and 78.1 (4.6) years in July 2020, compared with those not in the sample who were 69.9 (4.6) at baseline and 79.7 (4.5) in July 2020 (*p* < 0.001, respectively). The COVID-19 survey sample also had higher baseline education with 10.3 (3.5) years compared with 9.5 (3.4) among those not in the sample (*p* = 0.001). In the sample, those who responded were younger than those who did not (77.7 vs. 79.7 years in July 2020, *p* < 0.001) but did not differ in education (Table 1).

**Table 1:** Characteristics of the sample based on participation.

		**Respondents (*n* = 613)**		**Non-respondents (*n* = 246)^a^**		
		**Mean**	**(SD)**	**Mean**	**(SD)**	***P*-value^b^**
Age at FINGER baseline (years)		67.9	(4.6)	69.6	(4.7)	<0.001
Education (years)		10.2	(3.5)	10.1	(3.4)	0.572
Age at the time of COVID-19 questionnaire (years)^c^		77.7	(4.5)	79.5	(4.7)	<0.001
		***n***	**%**	***n***	**%**	
Women		299	48.8%	107	43.5%	0.161
Original intervention group		297	48.5%	135	54.9%	0.089
Living with someone at baseline		474	77.6%	173	70.9%	0.040
Living at capital area		213	34.7%	91	37.0%	0.534
Living with someone at the time of COVID-19 questionnaire		408	67.6%	n/a		
Chronic conditions (self-report)						
	0	123	20.4%	n/a		
	1	153	25.3%			
	2+	328	54.3%			

*COVID-19, coronavirus disease 2019; FINGER, Finnish Geriatric Intervention Study to Prevent Cognitive Impairment and Disability*.

a *Non-respondents as of September 2020; these data are preliminary, with the second data collection still ongoing from non-respondents*.

b *p-values from t-test for independent samples for continuous variables or χ^2^-test for categorical variables*.

c *Age calculated at the time of sending the questionnaire*.

Response rate was slightly higher among those who were living with someone (73 vs. 66%, *p* = 0.040). The majority (*n* = 577, 97%) of persons were able to answer themselves, while 18 (3%) needed help in completing the questionnaire or it was completed by someone else.

Data related to practicing distancing measures are presented in Table 2. The majority of participants (*n* = 458; 75%) reported practicing some social distancing, and the average duration of any type (or all types in total) was 9.2 weeks. At the time of completing the questionnaire, 354 (66%) reported still practicing some restrictions, with partial social distancing as the most common type (*n* = 255, 48%). Older participants (above median 77 years) reported any type of distancing less often than younger persons (71 vs. 79%, *p* = 0.025) but more often total isolation (29 vs. 19%, *p* = 0.003). Partial isolation was less common among older participants (47 vs. 63%, *p* < 0.001). Persons who lived alone were more likely to not do any social distancing (30 vs. 22%, *p* = 0.056), but there were no differences in specific types of distancing.

**Table 2:** Distancing measures during the first wave of the 2020 COVID-19 pandemic.

	**Participants reporting the type of distancing**		**Reporting duration**	**Average duration in weeks**	
	***n***	**%**	***n***	**Mean**	**(SD)**
No distancing	153	(25.0)			
Any distancing^a^	458	(75.0)	324	9.2	(3.9)
Total isolation (self-initiated or authority-enforced)	146	(23.9)	122	7.1	(4.0)
Social distancing	149	(24.4)	133	8.3	(3.7)
Partial social distancing	334	(54.7)	260	8.2	(4.0)

*COVID-19, coronavirus disease 2019*.

a *Participants reporting total isolation, social distancing, or partial distancing, or any combination of them. Duration calculated as a total of all types reported*.

The presence of chronic health conditions was reported by most participants (*n* = 481, 78%; number of diagnoses ranged from 1 to 7), with 54% of the study population reporting to have two or more chronic conditions (Table 1). Access to non-acute health care during the first phase of the COVID-19 pandemic is reported in Table 3. Approximately half the participants did not need care for their condition(s) during the first 4 months of the pandemic, and about one quarter had participated in a normal face-to-face visit. It was uncommon for health-care professionals to cancel appointments (~5% in the whole group with a condition and 10% among those who needed an examination). However, cancellation of other types of services was much more common; 109 (17% of the whole population, 45% of those who were due to have visit) reported some type of service being canceled by the professional, and 56 (9% in the whole sample; 23% among those in need) canceled the visits themselves. The most needed service that was more often canceled by the professionals was dental health care, but the proportion of canceled visits/help was almost equal in mental health care, social services, home aid, and rehabilitation (Table 3).

**Table 3:** Need and cancellation of non-acute health-care visits and other services during the first wave of the 2020 COVID-19 pandemic.

	**No need**		**Participant canceled**		**Professional canceled**		**Remote contact**^a^		**Normal appointment**	
	***n***	**%**	***n***	**%**	***n***	**%**	***n***	**%**	***n***	**%**
**Among those with at least one chronic condition**, ***n*** = **481^b^**										
Health-care visits related to non-acute chronic conditions	261	54.3	14	2.9	23	4.8	36	7.5	131	27.2
**Among all participants (proportions of cancellation types among those in need of a visit**, ***n*** = **6-205)**										
Dental health care	369	64.3	44	21.5	88	42.9	n/a		73	35.6
Mental health care	541	98.9	1	16.7	2	33.3	n/a		3	50
Social services	541	98.9	0	0	3	50	n/a		3	50
Home aid and services	530	96.4	4	20	6	30	n/a		10	50
Rehabilitative services and day services	496	90.3	12	22.6	28	52.8	n/a		13	24.5

*COVID-19, coronavirus disease 2019*.

a *This option was not provided for other types of care needed*.

b *Participants reported need and cancellation for each of the condition separately, and thus, the same participants may have reported several cancellation alternatives. Proportions are calculated using all participants who reported having conditions, not only among those who answered to the need and cancellation question, and thus do not sum up to 100%*.

**Figure 2:**
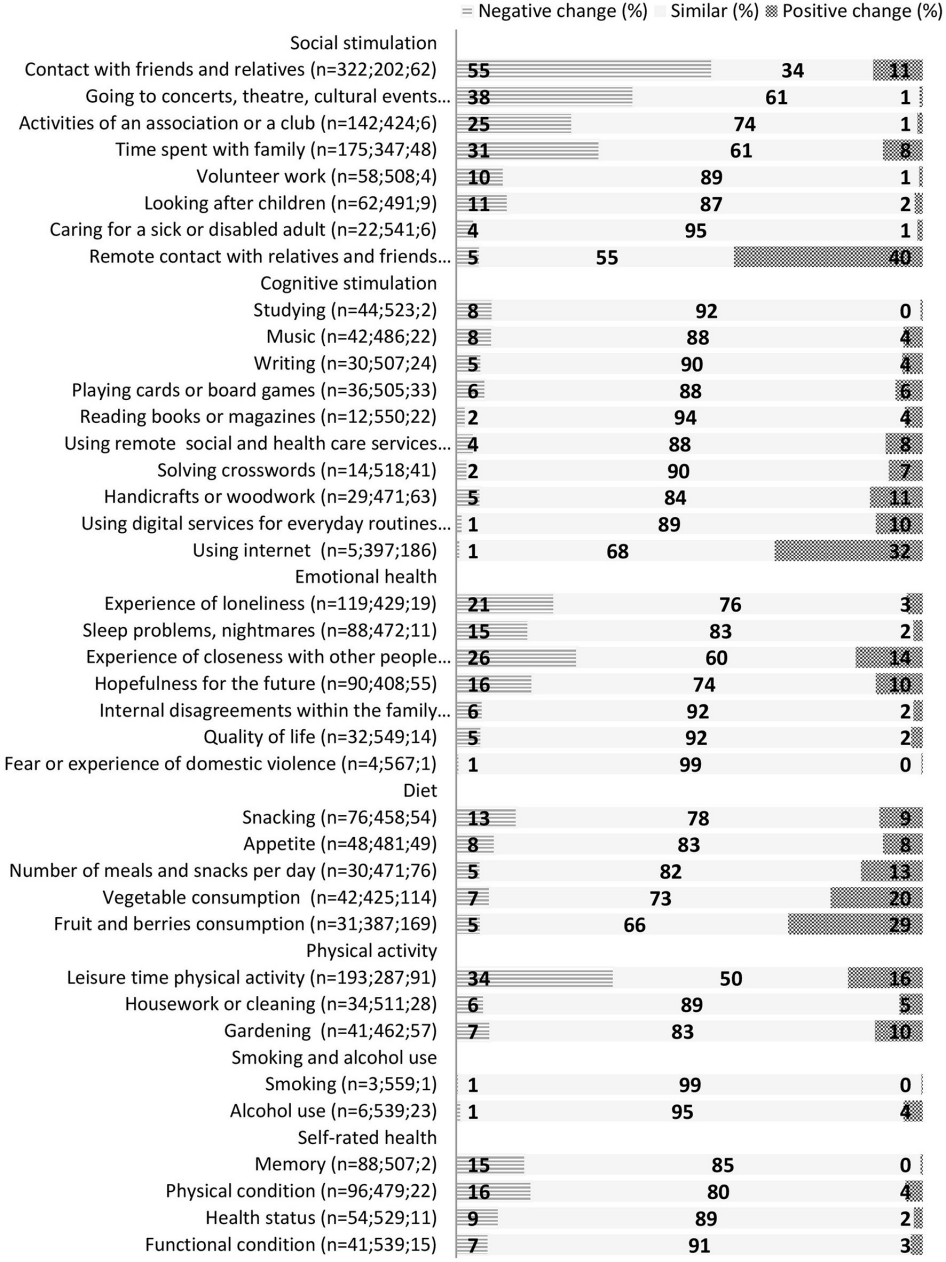
Self-evaluated changes in cognitive and social activities, emotional health, and lifestyle during the first wave of the 2020 COVID-19 pandemic.

Self-evaluated experiences of changes to aspects of daily life, lifestyle, social and cognitive activities, and self-rated health are presented in Figure 2. The items in the questionnaire are ranked according to the difference in negative and positive changes; that is, the items with the most negative changes (without a substantial amount of positive changes) are presented first, and items with most often reported positive change are reported last within each category. Most of the daily life and lifestyle-related items did not change substantially, especially alcohol consumption, smoking, and fear of domestic violence. Appetite changed in both directions equally. Remote contact with friends and using the internet in general was reported as having increased, both of which were considered as improvement. Time spent with family and contact with friends was reduced, as well as amount of physical activity. However, many participants reported doing more physical activity during the pandemic. Changes in diet were mostly positive, with increased consumption of both fruits and vegetables.

For changes in social and cognitive activities, participants mostly reported negative changes in attending cultural events such as concerts and theater, or meetings in clubs or societies. Voluntary work and looking after children also reduced during the pandemic. Few items in the hobby-type of activities had a positive change, but doing handicrafts was reported somewhat more often.

Self-rated health, physical functioning, memory, physical condition, and quality of life were relatively stable (Figure 2). There was a negative change in health, functioning, and quality of life in 9, 7, and 5% of respondents, respectively; and physical condition was evaluated as worsened for 16% and memory for 15% of the participants.

Older participants reported an increase in feelings of loneliness more often than younger participants (25 vs. 17%, *p* = 0.023), and a smaller proportion of older compared with younger participants reported that internet usage increased (24 vs. 39%, *p* > 0.001). Looking after children was more commonly reduced among the younger participants compared with older persons (15 vs. 7%, *p* = 0.004).

**Figure 3:**
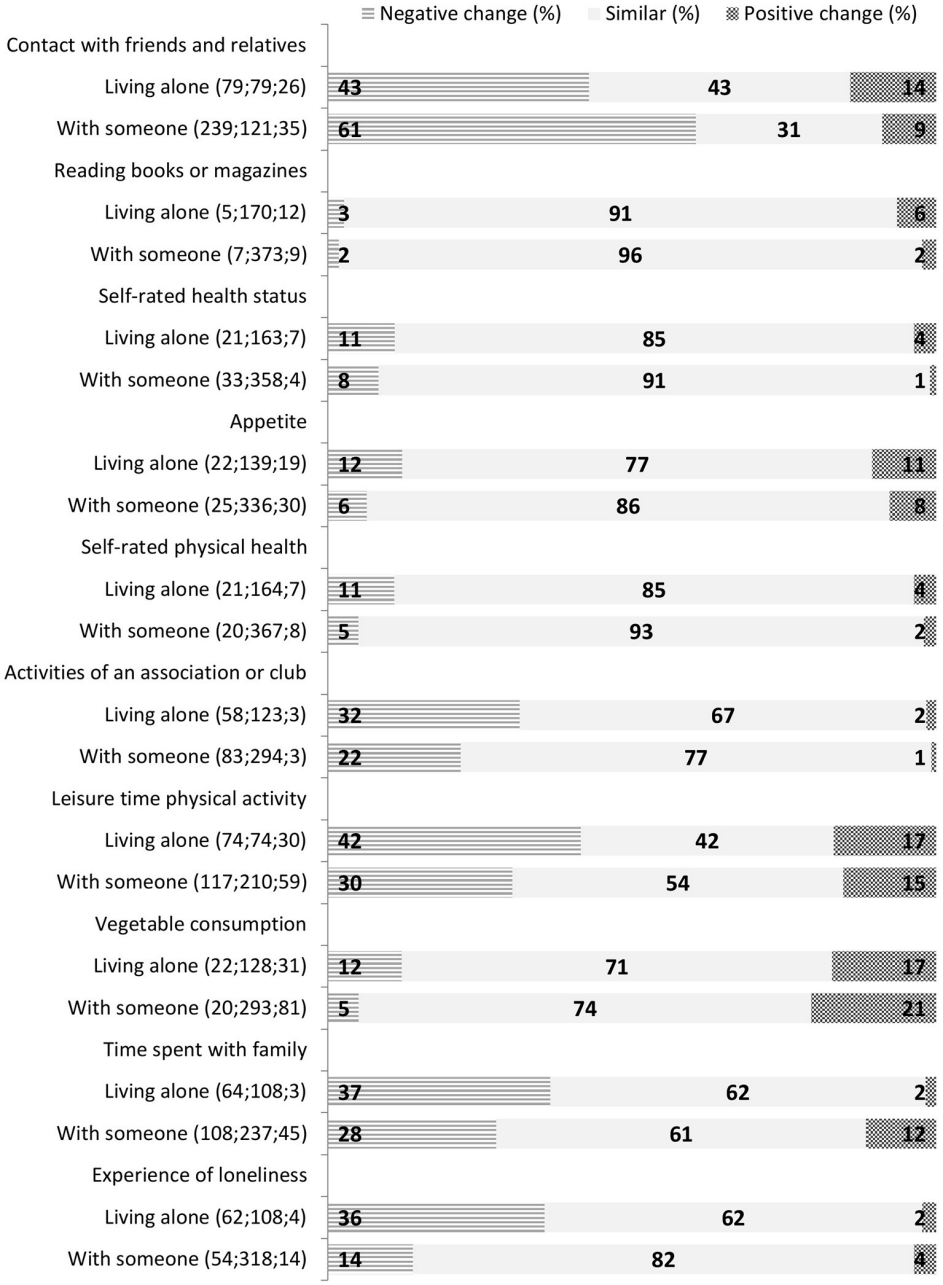
Self-evaluated changes in cognitive and social activities, emotional health, and lifestyle that were significantly (*p* < 0.05) different between those living alone or with someone.

All items from Figure 2 that are different between participants who lived alone or with someone are presented in Figure 3, ranked according to the difference between these group in negative and positive changes (the items with more favorable changes among those living alone are presented first, and the items with more negative changes among those living alone are last). Participants who lived alone reported a negative change in time spent with family more often (*p* < 0.001), but they reported decreased contact with friends less often (*p* < 0.001). Increased feelings of loneliness were more common (*p* > 0.001). They also reported more often a reduction in physical activity (*p* = 0. 013) and in vegetable intake (*p* = 0.024). Appetite was more often changed among those living alone, but changes were both positive and negative (*p* = 0.025). Those living alone more often reported a reduction in participating in clubs and societies (*p* = 0.026), and more increase in time spent reading (*p* = 0.036). They also reported a negative change in physical functioning more often than those who lived with others (*p* = 0.014) and more changes in self-rated health (both directions, proportion of similar as before *p* = 0.045).

## Discussion

Our survey reports lifestyle and health behaviors during the first wave of the COVID-19 pandemic (March–May 2020) in a sample of Finnish older adults who all were at risk of dementia, and some already had dementia. About three quarters of participants practiced social distancing measures, over an average of more than 9 weeks, with older participants more likely to practice total isolation than younger ones. Importantly, despite the relaxing of infection-control measures, about two thirds of participants were still practicing some social distancing measures when they sent their survey responses, mostly partial distancing. In terms of behaviors and factors that can affect cognitive decline, we found different patterns depending on the type of activity. Cognitively stimulating activities such as using the internet, doing handicrafts, and solving crosswords were largely unchanged or increased. However, attending concerts, theater, or other cultural events of participating in activities of clubs or associations decreased, as expected. In terms of diet, fruit and vegetable consumption mostly improved or remained unchanged. Leisure time physical activity was reduced for a third of the survey participants. Although most of the participants in the sample have one or more chronic health conditions; health care for chronic conditions was not hugely affected during the pandemic, with <10% missing planned health-care visits. Some behavior changes were more pronounced in older persons and those living alone, mostly the latter.

It is well-established that a combination of healthy lifestyle factors, including diet, physical exercise, opportunities for cognitive and social stimulation, and metabolic and vascular risk monitoring, is important for reducing risk of cognitive decline and disability (18, 19). The results of our study suggest that, mostly, these lifestyle factors did not change dramatically in our sample during the first wave of the COVID-19 pandemic. The most relevant change was a reduction in leisure time physical activity, as expected, because most sports and leisure facilities were closed. Another study in community-dwelling older adults in Japan also reported a decline in physical activity during the COVID-19 pandemic (6). However, it was a positive finding that many dietary aspects improved in our study population, including vegetable and fruit intake. In contrast, a study on Italian older persons with subjective cognitive deficits or mild cognitive impairment (MCI) found that they were more likely to engage in lifestyle changes that were potentially harmful to their future cognitive decline during compared with before the pandemic (20). Approximately half of this population underwent a dietary intervention as part of the FINGER trial and may, thus, have been more aware of the risks of unhealthy diets and possibly more likely to adopt healthier dietary changes during a period when they may have been worried about ill health as a result of being indoors. Furthermore, during lockdown, people were likely to spend more time at home and may have had more time for cooking.

As metabolic and vascular risk monitoring is an important element of multidomain interventions, we were interested to see whether participants experienced a reduction in access to health care. Only 10% of people who needed a medical appointment were unable to attend them because they had been canceled by the health-care professional. Interestingly, despite the Government action to cancel non-urgent health care, a quarter of our participants had a normal medical appointment during the first wave of the pandemic. This is in contrast with studies from other countries that report significant disruptions to non-acute, routine NCD care during the first wave of the pandemic (9, 10). However, the restrictions in Finland were gradually lifted in May–June, and there is a possibility that these appointments were postponed, although completed by the time of the questionnaire. A study from the Netherlands identified patients who are at risk of adverse outcomes of the corona measures, that is, discontinued care, social isolation, psychological, and behavioral problem (21).

The survey revealed some important results on social distancing. Although the Finnish Government made strong recommendations for social distancing, they did not enforce any rules. About 75% of participants practiced social distancing measures over an average of more than 9 weeks, which suggests that this sample was quite compliant with Government recommendations. Importantly, despite a relaxing of infection-control measures occurring in the summer, about two thirds of participants were still practicing some social distancing measures when they sent their survey responses (most persons returned their surveys in June–July).

Living status affected change in lifestyles, with those living alone more likely to report a reduction in time spent with family, although less likely with friends. They still reported increased feelings of loneliness, reduction in physical activity and physical functioning, and less improvement in vegetable intake than those who lived with others. More people who lived alone reported not following any social distancing measures, compared with people who live with others. A Japanese study reported that most patients with dementia or MCI who lived alone did not limit their outings or activities during the COVID-19 outbreak, but they were less likely to go out than healthy people (22). Older participants reported an increase in feelings of loneliness more often than younger participants, but no other major differences in age were found except a less increase in internet usage. A study from Germany and Poland reported that older people rated their quality higher of life, life satisfaction, and well-being during pandemic higher than did younger people (23).

Self-rated health, physical functioning, physical condition, and quality of life were relatively stable, but some emotional factors changed, interestingly, in both directions. For example, while there was a negative change in hopefulness for the future in 16% of people, 10% had a positive change. Similarly, although a quarter of people experienced less closeness with others, 14% had a positive change. This reflects how individual experiences of the COVID-19 pandemic can vary greatly. It will be interesting to establish which factors affect an individual's emotional response. For example, the CHARIOT COVID-19 Rapid Response Study reported that women; younger participants; and those who were single, widowed or divorced reported more feelings of loneliness and poor sleep, while those living alone were more likely to indicate poorer changes in depression and/or anxiety symptoms (24). Differences in levels of cognitive impairment are likely to also cause differences. A multicenter Italian study on outpatients with dementia concluded that infection-control policies such as quarantine can induce a rapid increase of behavioral and psychological symptoms of dementia (BPSD) in more than half of the patients (25).

Compared with the first results of the changes reported in the Finnish general population^5^,^6^, older adults in our study reported similar reduction in contacts with family and friends, with 62% having reduction in time spent with either family or friends. However, hopefulness was less often decreased in our population, and lifestyles such as in increases in snacking and any direction of changes in leisure time physical activity were less evident in this older population. It could be expected that everyday lives of older people, who are no longer working, are less affected by the pandemic than persons who are of working age and may tend to move outside their home more and engage more in activities with other people. However, restrictions in meeting family and friends may be more difficult to manage for older people, especially those living alone. It was evident in our results that while isolation is less adopted, consequences of restrictions may be more significant for those living alone.

The strengths of our study are that we conducted a survey in an already established research cohort, with a large sample, using a questionnaire designed within the World-Wide FINGERS Network (14), to allow future international comparisons. Response rate was high (71%), and the pre-pandemic data from previous waves of the FINGER project (since 2009) will allow for us to evaluate objective changes in health and lifestyle status in the future. However, the data are preliminary. Currently, we only have access to self-reported behavior change, whereas in the future, we will be able to compare with participants' previous comprehensive data from FINGER project. However, no other ethically acceptable alternatives than self-reports were available at the moment of the survey to avoid risk of infection of participants or staff via face-to-face interviews. The generalization of our results may be limited. First, there were some differences in respondents; those who sent back responses were younger and more likely to be living with someone. Second, the FINGER participants were originally selected due to their age and risk of developing cognitive impairment, and therefore, our results are only generalizable to this group of adults. There is also a potential selection bias because almost half the participants were part of the original FINGER intervention group, and therefore, they may be more aware of the importance of risk factor control and less likely to engage in negative health behaviors than other populations, even during the pandemic. Another potential limitation is that our survey was sent out to participants in June 2020, at the end of the first wave of the pandemic. Participants were asked to respond in terms of their behavior during the first wave of the pandemic, and therefore, their responses may have been less accurate because they had to recall their behaviors. Further, it is possible that behaviors may have changed over the period; some people may have been more active or engaged in more health behaviors in the beginning or vice versa. Therefore, we aimed to assess an overall change in behavior before and during the pandemic. Finally, we used simple statistical methods for comparing age groups and those living alone vs. not living alone, without adjusting for other covariates. These are preliminary descriptive data of the cohort, and we will later be able to combine these data with data collected from earlier FINGER follow-ups and also adjust for more covariates.

Results from our survey have relevant implications. Changes in health and lifestyle factors in older people may have important relevance for their long-term health and cognition by changing risk factors and, consequently, risk for future NCDs. Indeed, the findings of the FINGER intervention have highlighted the importance of multiple domains for preventing cognitive decline as well as other outcomes such as multimorbidity (4). Further, although we did not find many cancellations in routine NCD health-care appointments, it has been highlighted that changes in routine medical care, especially in older persons, will have a significant effect on risk factor management, potentially increasing risk of future NCDs and affecting mortality due to delays in diagnosis (26). Further, research into changes in lifestyle risk factors as a result of the pandemic are important, not only because these risk factors are relevant for NCDs but also because they can play a role in viral infections and viruses such as COVID-19. For example, a systematic review highlighted the importance of balanced nutrition for preventing and managing viral infections such as COVID-19, especially in older populations (27).

Europe is already undergoing a second wave, and new partial or total lockdown scenarios are already occurring in some countries and are likely in others. It is not possible to predict how long the COVID-19 pandemic will continue, and how often waves will reoccur. It is likely, therefore, that older individuals and those with NCDs, who are more likely to experience COVID-19 complications and related death (28, 29), will need to continue methods to shield themselves to avoid risk of SARS-CoV-2 infection in coming months or years. Therefore, it is imperative to understand how infection-control measures will affect lifestyle behaviors and NCD risk factors, and how to manage these in the short and long term. Future comparisons of country-specific policies and infection-control measures are therefore imperative to understand how people behave as a result of different measures. Indeed, it is interesting that the majority of participants in the current study practiced social distancing, despite no enforcement of recommendations by the Finnish Authorities. A recent Eurobarometer released in October 2020 suggested that Finnish people were one of the European nationalities that were more likely to report finding it easier to cope with confinement measures than other European countries^7^. Thus, cross-country comparisons focusing on specific factors that may influence differences in coping abilities may provide valuable insights.

### Future Research/Unanswered Questions

The current paper reports preliminary data from the FINGER COVID-19 survey. In the future, we plan to assess responses from the COVID-19 survey in relation to participants' pre-pandemic status. Further, as we developed the survey in collaboration with the WORLDWIDE-FINGERS-SARS-COV-2 INITIATIVE, it will be possible in the near future to make cross-country comparisons of how the COVID-19 pandemic affect older persons at risk of cognitive impairment in different settings. The survey is aligned with the WHO “Neurology and COVID-19 Global Forum,” which aims to enable harmonized approaches to clinical management, surveillance, and research on neurological disorders in the context of the COVID-19 pandemic. Future research should focus on individual characteristics that predict which people are most affected by lockdowns and infection-control policies, including differences according to age, sex, cognition, social support, living conditions, and access to outdoor space, among others. Further, an important avenue for future research is the possibility of developing and testing remote multidomain interventions (digital and telehealth) to replace face-to-face options during COVID-19 times.

## Conclusions

In conclusion, our survey of older persons in Finland at risk of cognitive impairment showed that there were less negatives changes in lifestyles and behaviors in this population than expected. However, age and living status may affect changes in risk factors that can increase the risk of cognitive decline and other negative outcomes such as disability and mortality.

## Data Availability Statement

The datasets presented in this article are not readily available because Public deposition of the de-identified data set is not possible due to legal and ethical reasons, and complete de-identification is not possible as this investigation is part of an ongoing study. The study participants gave informed consent which includes data use only under confidentiality agreement. Further, the data contains large amount of sensitive information and public data deposition may pose privacy concerns. Those fulfilling the requirements for viewing confidential data as required by the Finnish law and the Finnish Institute for Health and Welfare are able to access the data after completion of material transfer agreement. Requests to access the datasets should be directed to kirjaamo@thl.fi.

## Ethics Statement

The studies involving human participants were reviewed and approved by Coordinating Ethics Committee of the Hospital District of Helsinki and Uusimaa, Finland. The patients/participants provided their written informed consent to participate in this study.

## Author Contributions

MK, TN, and AS devised the study objectives. MK, TN, AS, JL, FM, and KP designed the survey and interpreted the results. MK, TN, JL were responsible for data collection. JL and KP conducted the data analysis and results and wrote the article. MK, TN, AS, and FM critically revised the manuscript. All authors contributed to the article and approved the submitted version.

## Conflict of Interest

The authors declare that the research was conducted in the absence of any commercial or financial relationships that could be construed as a potential conflict of interest.

## Footnotes

^1^Available online at: https://www.who.int/mental_health/neurology/dementia/guidelines_risk_reduction/en/ (accessed October 23, 2020).

^2^Available online at: THL/National Infectious Disease Register. https://experience.arcgis.com/experience/92e9bb33fac744c9a084381fc35aa3c7 (accessed 23/10/2020).

^3^Available online at: https://sampo.thl.fi/pivot/prod/en/epirapo/covid19case/fact_epirapo_covid19case (accessed January 27, 2020).

^4^Available online at: https://thl.fi/en/web/thlfi-en/research-and-expertwork/projects-and-programmes/serological-population-study-of-the-coronavirus-epidemic (accessed October 30, 2020)

^5^Available online at: https://thl.fi/en/web/thlfi-en/-/the-coronavirus-epidemic-has-reduced-social-interaction-and-the-use-of-services-impact-on-lifestyles-as-well (accessed 30/10/2020).

^6^Available online at: https://www.julkari.fi/handle/10024/139889 (accessed 30/10/2020).

^7^Available online at: https://ec.europa.eu/commfrontoffice/publicopinion/index.cfm/survey/getsurveydetail/instruments/standard/surveyky/2262 (accessed October 30, 2020).
